# Supplementary material for: Discovery and pharmacophoric characterization of chemokine network inhibitors using phage-display, saturation mutagenesis and computational modelling
Source: Nat Commun. 2023 Sep 16;14:5763. doi: 10.1038/s41467-023-41488-z (PMC10505172; doi:10.1038/s41467-023-41488-z)
Supplement: Supplementary file 4 — Source Data [file 41467_2023_41488_MOESM4_ESM.zip › SourceData/scripts/xdock_20230808165437.tar!/xdock/analysis/SV_msa.pdf]

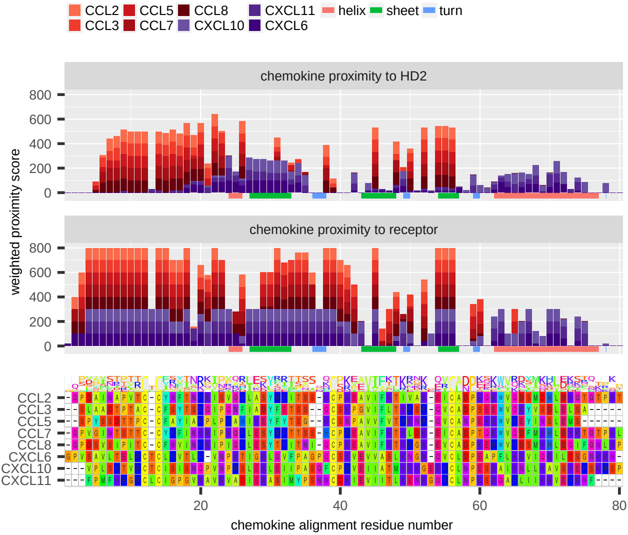

CCL2
CCL3
CCL5
CCL7
CCL8
CXCL10
CXCL11
CXCL6
helix
sheet
turn
chemokine proximity to HD2
chemokine proximity to receptor
weighted proximity score
0
200
400
600
800
CCL2
CCL3
CCL5
CCL7
CCL8
CXCL6
CXCL10
CXCL11
20
40
60
80
chemokine alignment residue number
